# Association between Malnutrition and 28-Day Mortality and Intensive Care Length-of-Stay in the Critically ill: A Prospective Cohort Study

**DOI:** 10.3390/nu10010010

**Published:** 2017-12-23

**Authors:** Charles Chin Han Lew, Gabriel Jun Yung Wong, Ka Po Cheung, Ai Ping Chua, Mary Foong Fong Chong, Michelle Miller

**Affiliations:** 1 Discipline of Nutrition and Dietetics, School of Health Sciences, Flinders University, GPO Box 2100, Adelaide SA 5001 Australia; michelle.miller@flinders.edu.au; 2 Dietetics and Nutrition Department, Ng Teng Fong General Hospital, Singapore City 609606, Singapore; gabriel_wong@nuhs.edu.sg (G.J.Y.W.); kpcheungapril@gmail.com (K.P.C.); 3 Department of Respiratory Medicine, Ng Teng Fong General Hospital, Singapore City 609606, Singapore; ai_ping_chua@nuhs.edu.sg; 4 Saw Swee Hock School of Public Health, National University of Singapore, Singapore City 119077, Singapore; mary_chong@nus.edu.sg

**Keywords:** malnutrition, nutritional assessment, mortality, length-of-stay, critical illness

## Abstract

There is limited evidence for the association between malnutrition and mortality as well as Intensive Care Unit length-of-stay (ICU-LOS) in critically ill patients. We aimed to examine the aforementioned associations by conducting a prospective cohort study in an ICU of a Singapore tertiary hospital. Between August 2015 and October 2016, all adult patients with ≥ 24 h of ICU-LOS were included. The 7-point Subjective Global Assessment (7-point SGA) was used to determine patients’ nutritional status within 48 h of ICU admission. Multivariable regressions were conducted in two ways: (1) presence versus absence of malnutrition, and (2) dose-dependent association for each 1-point decrease in the 7-point SGA. There were 439 patients of which 28.0% were malnourished, and the 28-day mortality rate was 28.0%. Malnutrition was associated with an increased risk of 28-day mortality (adjusted Relative Risk 1.33 (95% Confidence Interval: 1.05–1.69)), and this risk increased with a greater degree of malnutrition (adjusted Relative Risk 1.08 (95% Confidence Interval: 1.00–1.16) for each 1-point decrease in the 7-point SGA). No significant association was found between malnutrition and ICU-LOS. The results of this study indicated a clear association between malnutrition and higher 28-day mortality in critically ill patients. The association between malnutrition and ICU-LOS could not be replicated and hence requires further evaluation.

## 1. Introduction

Malnutrition within the critical care setting is a global issue where prevalence in developing and developed countries can be as high as 78.1% and 50.8%, respectively [1]. Lew et al. [1] recently conducted a systematic review to determine the association between malnutrition and worsened clinical outcomes in the Intensive Care Unit (ICU). Two nutrition assessment and 10 nutrition screening tools were identified in the review. The recent European Society for Clinical Nutrition and Metabolism malnutrition diagnostic criteria [2] and the Academy of Nutrition and Dietetics/American Society for Parenteral and Enteral Nutrition malnutrition clinical characteristics [3] were not included, since their validity in the ICU have not been measured. The review demonstrated that nutrition risk determined by nutrition screening tools (e.g., Nutritional Risk Screening-2002 [4], Malnutrition Universal Screening Tool [5]) showed inconsistent association with clinical outcomes [1]. On the contrary, malnutrition diagnosed by the Subjective Global Assessment (SGA) [6] was consistently associated with increased length-of-stay in the ICU and a higher risk of mortality. Therefore, the systematic review recommended the use of the SGA in the critical care setting [1].

The systematic review also identified possible limitations in the included primary studies. For example, there was a lack of blinding of treatment team (i.e., intensivists and nurses) to the objective of the studies. This may introduce treatment bias that weakens the validity of the association between malnutrition and worsened clinical outcomes in the ICU [1].

Another evaluation of the primary studies included in the systematic review [1] is the quality of statistical adjustment, as optimal statistical adjustment is essential for a valid quantification of the association between a particular risk factor and the outcome of interest [7]. The primary studies used the Acute Physiologic and Chronic Health Evaluation II (APACHE II) crude score [8] instead of the predicted mortality risk (PMR) to adjust for mortality risk. This may not be ideal because the PMR better reflects actual mortality risk by factoring both the admission diagnosis and the APACHE II crude score in its derivation [8]. Consequently, the APACHE II crude score of patients with different admission diagnoses can be identical, yet the PMR may differ due to the difference in mortality associated with the diagnoses [8,9,10]. For example, patients with congestive heart failure and an APACHE II score of 23 would have a PMR of 36%. In contrast, the same APACHE II score would translate to a PMR of 64% in patients with sepsis. Therefore, the PMR may be a more appropriate covariate for statistical adjustment of mortality risk than the APACHE II crude score.

In response to the systematic review conducted by Lew et al. [1], which highlighted considerable limitations in the included studies, such as sub-optimal statistical adjustment, and the lack of blinding and dose-dependent analysis, this study aimed to overcome these limitations in an effort to perform a valid determination of the association between malnutrition and 28-day mortality and ICU length-of-stay (ICU-LOS) amongst critically ill patients.

## 2. Materials and Methods

This prospective observational cohort study was conducted in the ICU of Ng Teng Fong General Hospital (Singapore). Between August 2015 and October 2016, consecutive patients admitted to the ICU were screened for eligibility. Patients ≥18 years old who had ≥ 24 h ICU-LOS were enrolled, and only data from their first ICU admission within the same hospitalization were included in the study. The physicians and nurses were blinded to the objective of the study to reduce the risk of selection and treatment biases. The Domain Specific Review Board approved this study (NHG DSRB Ref: 2014/00878) and informed consent was not required since this was an observational study where no attempt was made to change the standard of care. This study is registered with ClinicalTrials.gov, number NCT03213899, and the reporting of this study followed the TRIPOD statement [11].

### 2.1. Data Collection

The ICU contains 35 beds and functions as a closed unit that provides support to both medical and surgical patients. The unit also concurrently functions as a High Dependency (HD) Unit, as patients’ status can be changed between ICU-status and HD-status within the same ICU-/HD-bed. Patients are classified as “ICU-status” when mechanically ventilated and requiring support of two or more organ systems. They are downgraded to HD-status once they are extubated from mechanical ventilation. When in HD-status, patients are treated by the same physicians and allied health professionals. The only difference between ICU- and HD-status is the nursing to patient ratio, where it changes from 1:1 to 1:2, respectively. 

All data were prospectively measured and recorded in the electronic medical records. The primary outcomes were 28-day mortality and ICU-LOS. For ICU-LOS (in days), duration was measured from the date of the first ICU admission to the date of the first change in ICU-status to HD-status or discharge to the general ward. To enable robust statistical adjustments, other parameters known to be covariates for mortality and ICU-LOS [12,13] were also collected (i.e., location, length of hospitalization, and presence/absence of vasoactives and cardiopulmonary resuscitation before ICU admission; APACHE II; PMR derived from the APACHE II and admission diagnosis [8]; Sequential Organ Failure Assessment (SOFA) [14]; Charlson Comorbidity Index [15]; length of mechanical ventilation; and ICU and hospital length-of-stay). 

### 2.2. Nutrition Assessment

It is part of routine care for all ICU patients to receive nutrition assessment (i.e., 7-point SGA) within 48 h of admission to the ICU. Three experienced dietitians performed the 7-point SGA and agreement between the dietitians was previously measured in 68 patients. The weighted kappa was 0.85 (standard error = 0.079, *p*-value < 0.001), indicating good agreement. Information required for the 7-point SGA was obtained from either the patients or their main caregivers. In cases where nutritional status could not be determined within the first 48 h (due to inadequate information), data on nutritional status were considered as “missing”. This was to minimize reverse causality bias, as the study aimed to determine the association between premorbid malnutrition and hospital mortality. 

The 7-point SGA [16,17] is a variant of the SGA [6]. It was used not only to determine the association between malnutrition and hospital mortality and ICU-LOS, but further allow a dose-dependent analysis. One key advantage for using the 7-point SGA is the detailed response options that improve the standardization and objectivity in the classification of nutritional status [17]. Similar to the conventional SGA, the 7-point SGA classifies nutritional status into three major categories (i.e., well-nourished, mildly-moderately malnourished, severely malnourished). Specifically, patients with SGA-A7 and SGA-A6 are well-nourished; SGA-B5, SGA-B4, and SGA-B3 are mildly-moderately malnourished; and SGA-C2 and SGA-C1 are severely malnourished. Each 1-point decrease reflects a greater degree of malnutrition, and this increased resolution allowed the association between malnutrition and hospital mortality to be analyzed in a dose-dependent manner.

### 2.3. Statistical Analysis

Patient characteristics were reported as mean and standard deviation (continuous variables) or counts and percentages (categorical variables) and were compared using Student’s *t*-test or Chi-square test, as appropriate. Medians and inter-quartile range were reported for variables that deviate from normality, and the Mann-Whitney U-test was used for comparison. The relative risk for the association between malnutrition (SGA-B5 to SGA-C1) and 28-day mortality was quantified using a modified Poisson regression model incorporating the robust sandwich variance [18]. Collinear variables (i.e., APACHE II and SOFA) were excluded and backward elimination of covariates was performed to obtain a parsimonious model. The dose-dependent relationship between the degree of malnutrition and 28-day mortality was quantified using the same Poisson regression with the exception of having nutritional status (SGA-A7 to SGA-C1) analyzed as a continuous variable. 

To explore the effects of sub-optimal statistical adjustment, two logistic regression models were compared. Model A contained commonly used covariates (i.e., age, duration of mechanical ventilation, APACHE II, and duration of stay in the ICU and hospital), while Model B contained all the above covariates, but replaced the APACHE II with PMR, and included additional covariates that are associated with ICU clinical outcomes but were often not adjusted in other studies (i.e., the presence/absence of vasoactive drugs and length of hospitalization before ICU admission). The McFadden’s pseudo-R^2^ and Akaike information criterion revealed that Model B performed better in that the McFadden’s pseudo-R^2^ and the Akaike information criterion of Model B were respectively 8.5% higher (43.0% versus 34.5%) and 40 units lower (315 versus 355) than Model A. Therefore, compared to the APACHE II, the PMR was a more appropriate covariate for statistical adjustment for mortality risk.

The association between malnutrition and ICU-LOS was determined by a series of simple linear regressions. Only ICU survivors were considered in the analysis to account for the competing risk of death on ICU-LOS [19]. Since the simple linear regressions showed that none of the patient characteristics were significantly associated to ICU-LOS, a multivariable linear regression was not carried out. Statistical analyses were performed using STATA 14.2 (Stata Corp, College Station, TX, USA) and significance was assumed at *p* < 0.05.

## 3. Results

**Figure 1:**
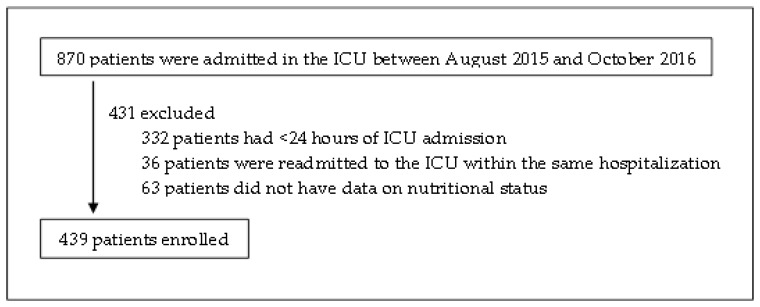
Enrollment of patients. ICU, Intensive Care Unit.

There were 502 eligible patients, but 63 were excluded as they lacked 7-point SGA data (Figure 1). Excluded patients had significantly shorter lengths of hospitalization (median: 8.0 days versus 14.0 days), less severe comorbidities (median of Charlson morbidity index: 0.0 versus 1.0), and proportionally less of them were admitted from the general wards (7.9% versus 18.7%). Amongst the remaining 439 patients (medical: 294, surgical: 145), sepsis (23.9%), respiratory (22.1%), neurological (22.1%), and cardiovascular (18.5%) conditions were the most common reasons for ICU admission. The 28-day mortality rate was 28.0% (*n* = 123), and no patients were lost to follow-up. 

Prevalence of malnutrition was 28% (mildly-moderately malnourished: 25% (SGA-B5: 13.4%, SGA-B4: 7.3%, SGA-B3: 4.3%), severely malnourished: 3% (SGA-C2: 2.7%, SGA-C1: 0.2%)). Malnourished patients were significantly older, had lower body mass index (BMI), and exhibited higher disease severity as compared to their well-nourished counterparts (Table 1). In addition, the prevalence of malnutrition was highest in patients admitted with sepsis (38.1%) and lowest in patients with neurological conditions (14.4%). Patients with respiratory and cardiovascular conditions had similar prevalence (24.7% and 28.4%, respectively).

Malnutrition was associated with a 33% increased risk of 28-day mortality. The dose-dependent analysis revealed that each 1-point decrease in the 7-point SGA (indicative of a greater degree of malnutrition) was associated with an 8% increase in the risk of 28-day mortality (Table 2). 

There were 363 patients who survived their ICU admission, and their median ICU-LOS was 2.0 days (interquartile range: 1.0, 5.0). Simple linear regression did not identify any covariate that was associated with ICU-LOS (Table 3). 

## 4. Discussion

To our knowledge, this is the largest study that used a validated nutrition assessment tool in an attempt to demonstrate an association between malnutrition and 28-day mortality and ICU-LOS amongst the critically ill. In addition, this is the first study that explored their relationships in a dose-dependent manner, which strengthened the findings.

The results of the present study could not be compared with those in previous studies, as only the odds ratio [20] or adjusted *p*-value [21] were reported. Nevertheless, malnutrition was consistently demonstrated to be associated with increased mortality risk. Since one of the rationales for limiting life-sustaining treatments in the ICU is poor prognosis, the clear association between malnutrition and increased mortality suggests that nutritional status should be considered along with other conventional prognostic parameters to aid treatment decisions. 

No significant association was found between malnutrition and ICU-LOS. This could be due to the short ICU-LOS where any association with malnutrition and other parameters (including disease severity) would be difficult to establish. The median ICU-LOS in the present study was notably shorter than a similar cohort in another local tertiary hospital (two versus four to five days) [22]. This could be due to the unique integration of the ICU/HD unit in the hospital that allows our ICU patients to quickly transit to HD care without a need to change location. It is likely a more accurate reflection of the required ICU-LOS as compared to other tertiary hospitals where ICU patients may need to wait for a physical bed in the HD unit before transfer and this may potentially inflate their ICU-LOS. Sheean et al. [21] also did not observe any association between malnutrition and ICU-LOS, and this may also be attributed to the relatively short mean ICU-LOS (i.e., three days). These findings are in contrast with the study by Caporossi et al. [23] where malnutrition was reported to be associated with prolonged ICU admission (mean ICU-LOS: nine days).

The present study further widened the range of malnutrition prevalence reported in the literature. In a recent systematic review [1], the prevalence of malnutrition amongst ICUs that admit heterogeneous types of patients was 38% to 78%, whereas the prevalence was 28% in the present study. The wide variability calls for studies in individual ICUs to determine their local malnutrition prevalence, and identify an appropriate nutrition screening tool (e.g., Nutritional Risk Screening-2002 [4]) to be used in their respective ICUs. These studies may use the SGA as the reference criterion, since the validity and reliability of the SGA in the ICU has been well demonstrated [1,24].

Compared to previous studies, this study has some strengths. First, the results are more generalizable with the inclusion of both medical and surgical patients. Second, measures were taken to reduce the risk of selection, attrition, treatment, and reverse causality biases. Thirdly, instead of computing the odds ratio, this study also expressed the strength of the association between malnutrition and hospital mortality in relative risk. This is important, as the prevalence of malnutrition was more than 10%, and the use of odds ratio will result in an overestimation of the association [25]. There are, however several limitations that deserve consideration. Firstly, some patients were excluded from the study due to missing 7-point SGA data. Although they had several characteristics that were significantly different from those patients with 7-point SGA data, these characteristics were either not associated with 28-day mortality and ICU-LOS, or they were adjusted using the multivariable models. Secondly, despite robust statistical adjustments, there remained a possibility of residual confounding in all observational studies. 

### 4.1. Future Research

As with Fontes et al. [20], it was beyond the scope of the present study to measure the extent of nutrition support rendered to both well- and malnourished patients. It is plausible that variations in the degree of nutrition support may modify the association between malnutrition and mortality. The corollary of this view is the question “will adequate nutrition support attenuate the mortality risk of malnourished patients in the ICU?” The optimal nutrition support strategy in the ICU (i.e., permissive underfeeding vs. meeting estimated energy requirements) remain nebulous and current evidence from randomized controlled trials is mixed [26]. A common limitation amongst the studies is the lack of baseline nutrition assessment, since it is conceptually possible that malnourished patients require more calories and protein to attenuate the deleterious effects of critical illness as compared to well-nourished patients [4,27]. Given the clear association between malnutrition and 28-day mortality, future studies that aim to determine the optimal nutrition support strategy for the critically ill should conduct nutrition assessment at baseline to better elucidate how nutritional status can modify the therapeutic effects of different feeding strategies.

## 5. Conclusions

There was clear evidence that malnutrition is independently associated with increased risk of 28-day mortality. This suggests that nutritional status, along with other conventional prognostic factors, should be considered to better predict 28-day mortality. The association between malnutrition and ICU-LOS, however, was not demonstrated in the present study. More studies are recommended to further evaluate this possible association. In addition, the prevalence of malnutrition in the present study was lower than those reported in a recent systematic review [1]. This highlighted the importance for individual ICUs to measure their local prevalence in order to guide their nutrition screening and assessment policies. Lastly, the present study provided a rationale for future studies to determine the interaction between baseline nutritional status and optimal goal of nutrition support on mortality outcomes.

## Figures and Tables

**Table 1:** Comparison of characteristics between well-nourished and malnourished patients, and 28-day survivors and non-survivors.

Parameters	Well-Nourished	Malnourished	*p*-Value	Survivor	Non-Survivor	*p*-Value
	(*n* = 316)	(*n* = 123)		(*n* = 316)	(*n* = 123)	
Age (years)	59.8 (15.7)	65.6 (15.3)	0.001	59.3 (15.9)	66.8 (14.1)	< 0.001
Male	188 (59.5)	69 (56.1)	0.517	191 (60.4)	67 (54.5)	0.254
BMI (kg/m^2^)	26.2 (5.8)	22.6 (5.8)	<0.001	25.2 (6.0)	25.1 (6.2)	0.960
Location before adm						
ED/HD/OT	263 (83.2)	94 (76.4)	0.100	268 (84.8)	89 (72.4)	0.003
Wards	53 (16.8)	29 (23.6)		48 (15.2)	34 (27.6)	
Type of adm						
No surgery	210 (66.5)	83 (67.5)	0.974	199 (63.0)	94 (76.4)	0.027
Elective surgery	10 (3.2)	4 (3.3)		11 (3.5)	3 (2.4)	
Emergency surgery	96 (30.4)	36 (29.3)		106 (33.5)	26 (21.1)	
Charlson morbidity index	1.0 (0.0, 3.0)	1.0 (1.0, 3.0)	0.054	1.0 (0.0, 3.0)	1.0 (0.0, 3.0)	0.320
LOS before ICU adm (days)	0.0 (0.0, 1.0)	1.0 (0.0, 3.0)	<0.001	0.0 (0.0, 1.0)	1.0 (0.0, 3.0)	0.008
APACHE II	23.7 (8.0)	26.9 (7.9)	<0.001	22.6 (7.4)	29.5 (7.7)	<0.001
SOFA	8.3 (3.6)	9.5 (4.2)	0.009	7.9 (3.5)	10.7 (3.8)	<0.001
Predicted mortality risk (%) ^a^	47.7 (25.8)	59.7 (24.9)	<0.001	44.1 (24.2)	68.8 (22.2)	<0.001
Vasoactives before ICU adm	134 (42.4)	59 (48.0)	0.292	127 (40.2)	66 (53.7)	0.011
CPR before ICU adm	35 (11.1)	18 (14.6)	0.304	17 (5.4)	36 (29.3)	<0.001
Length of MV (days)	2.0 (1.0, 5.0)	2.0 (1.0, 5.0)	0.734	2.0 (1.0, 4.0)	3.0 (2.0, 6.0)	<0.001
ICU LOS (days)	2.0 (2.0, 5.0)	3.0 (2.0, 5.0)	0.981	2.0 (1.0, 4.0)	3.0 (2.0, 6.0)	0.001
Hospital LOS (days)	13.0 (6.3, 24.0)	16.0 (9.0, 27.0)	0.120	15.0 (9.0, 33.0)	9.0 (4.0, 16.0)	<0.001
28-day mortality	72 (22.8)	51 (41.5)	<0.001			
Malnutrition				72 (22.8)	51 (41.5)	<0.001
SGA Sub-Categories						
SGA-7	217 (68.7)			165 (52.2)	52 (42.3)	
SGA-6	99 (31.3)			79 (25.0)	20 (16.3)	
SGA-5		59 (48.0)		38 (12.0)	21 (17.1)	
SGA-4		32 (26.0)		16 (5.1)	16 (13.0)	
SGA-3		19 (15.4)		9 (2.8)	10 (8.1)	
SGA-2		12 (9.8)		9 (2.8)	3 (2.4)	
SGA-1		1 (0.8)		0 (0.0)	1 (0.2)	

Values are mean (SD), median (q1, q3), or counts [percentage]. ^a^ derived from the Acute Physiologic and Chronic Health Evaluation II. adm, admission; APACHE II, Acute Physiology and Chronic Health Evaluation II; BMI, Body Mass Index; CPR, Cardiopulmonary Resuscitation; ED, Emergency Department; HD, High Dependency; ICU, Intensive Care Unit; LOS, Length-of-Stay; MV, Mechanical Ventilation; OT, Operation Theatre; SGA, Subjective Global Assessment; SOFA, Sequential Organ Failure Assessment.

**Table 2:** Multivariable analysis of the association between malnutrition and 28-day mortality.

Parameters	Risk Estimates ^a^		*p*-Value
Malnourished ^b^	Crude RR	1.82 (95%CI: 1.36, 2.44)	<0.001
	Adj-RR	1.33 (95% CI: 1.05, 1.69)	0.019
Every 1-point decrease in the 7-point SGA ^c^	Crude RR	1.18 (95%CI: 1.08, 1.30)	<0.001
	Adj-RR	1.08 (95% CI: 1.00, 1.16)	0.039

**^a^** adjusted for age; presence/absence of vasoactive drugs, and length of hospitalization before admission to the intensive care unit; duration of mechanical ventilation; predicted mortality risk derived from the Acute Physiologic and Chronic Health Evaluation II; and duration of stay in the intensive care unit and hospital; **^b^** Reference: Well-nourished (SGA-A7 or SGA-A6); **^c^** Every 1-point decrease is indicative of a higher degree of malnutrition, Adj, Adjusted; RR, Relative risk; CI, Confidence interval; SGA, Subjective global assessment.

**Table 3:** Simple linear regression models of the association between patient characteristics and length-of-stay in the Intensive Care Unit (measured in days) amongst patients who were discharged alive from the Intensive Care Unit.

Patient Characteristics (*n* = 363)	Standardized Beta Weight	95% Confidence Interval	*p*-Value
Age (years)	−0.100 ^b^	−0.105, 0.001	0.057
BMI (kg/m^2^)	0.052 ^b^	−0.072, 0.220	0.318
Admitted from the wards	0.005	−2.247, 2.479	0.923
No surgery	−0.200	−1.875, 1.230	0.700
Charlson morbidity index	−0.100 ^b^	−0.870, 0.011	0.056
LOS before ICU admission (days)	0.001 ^b^	−0.180, 0.182	0.063
APACHE II	−0.025 ^b^	−0.140, 0.085	0.632
SOFA	0.031 ^b^	−0.175, 0.324	0.559
Predicted Mortality Risk (%) ^a^	−0.042 ^b^	−0.049, 0.020	0.424
Given vasoactives before ICU admission	0.001	−1.726, 1.745	0.991
Given CPR before ICU admission	0.006	−2.980, 3.364	0.905
Length of MV (days)	0.068 ^b^	−0.213, 5.825	0.068
Malnutrition	−0.015	−2.245, 1.665	0.771

^a^ derived from the Acute Physiologic and Chronic Health Evaluation II; ^b^ every unit increase, APACHE II, Acute Physiologic and Chronic Health Evaluation II; BMI, Body Mass Index; CPR, Cardiopulmonary Resuscitation; ICU, Intensive Care Unit; LOS, Length-of-Stay; MV, Mechanical Ventilation; SOFA, Sequential Organ Failure Assessment.
